# Improvement of postharvest quality, secondary metabolites, antioxidant activity and quality attributes of *Prunus persica* L. subjected to solar drying and slice thickness

**DOI:** 10.1016/j.sjbs.2023.103866

**Published:** 2023-11-03

**Authors:** Hossam S. El-Beltagi, Ayesha Khan, Syed Tanveer Shah, Abdul Basit, Muhammad Sajid, Muhammad Hanif, Heba I. Mohamed

**Affiliations:** a Agricultural Biotechnology Department, College of Agriculture and Food Sciences, King Faisal University, Al-Ahsa 31982, Saudi Arabia; b Department of Horticulture, The University of Agriculture, Peshawar, Khyber Pakhtunkhwa, Pakistan; c Department of Agriculture, Faculty of Biological and Health Sciences, Hazara University, Mansehra, Khyber Pakhtunkhwa, Pakistan; d Department of Horticultural Science, Kyungpook National University, 41566 Daegu, South Korea; e Department of Agricultural Mechanization and Renewable Energy Technologies, The University of Agriculture Peshawar-Pakistan, Pakistan; f Department of Biological and Geological Sciences, Faculty of Education, Ain Shams, University, Cairo 11341, Egypt

**Keywords:** Antioxidant, Ascorbic acid, Drying methods, Flavonoids, Phenolic, *Prunus persica*

## Abstract

Peach is a fruit highly appreciated by consumers; however, it is highly perishable, so drying is an alternative to preserve its physical and chemical properties. In this study, the effect of different drying methods (oven, solar, and open sun) and slice thicknesses (0.5, 1.0, and 1.5 cm) on quality, shelf life, color, total phenol, flavonoid, reducing sugar, non reducing sugar, ascorbic acid contents and antioxidant capacity of peach (*Prunus pe*rsica L. Cv. Indian blood red), were evaluated. The results showed that, 0.5 cm slice thickness recorded the best results with minimum moisture content (%), drying time (7 hrs), titratable acidity (%), reducing sugars (%), non-reducing sugars (%), total soluble solids (°Brix), disease incidence (%), drying rate, TSS-Acid ratio, ascorbic acid, color and flavor, and total phenolic contents. Storage conditions showed significant results on 90 days of storage with maximum total soluble solids (°Brix), TSS-Acid ratio, reducing sugars (%), minimum titratable acidity (%), ascorbic acid, disease incidence (%), and non-reducing sugars (%), while color and flavor also retained. Peach fruits sliced at 0.5 cm thickness and dried in a solar collector may be considered best to retain the bio chemical attributes for 90 days and solar collector considered as environmentally friendly technology.

## Graphical abstract

**Figure:**
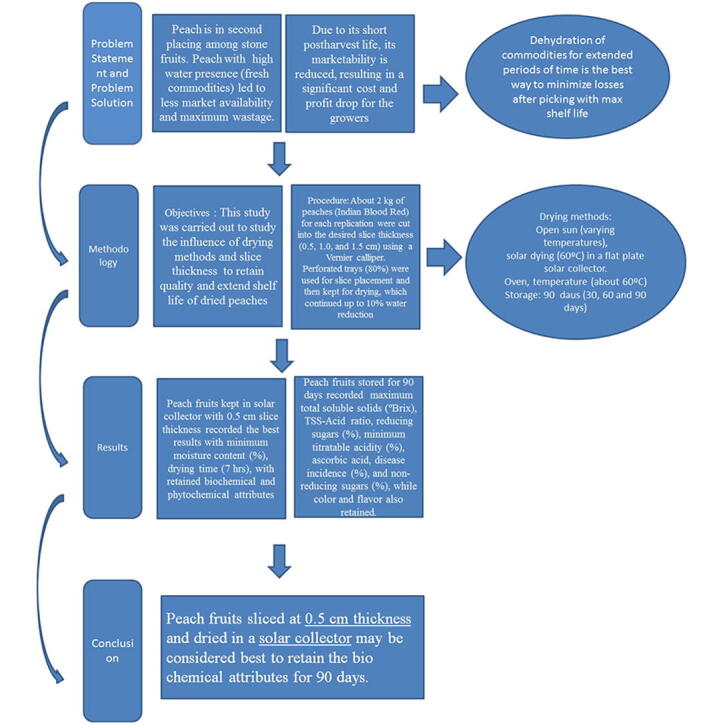

## 1 Introduction

Peach (Prunus persica L.) belongs to the Rosaceae family and is believed to be an inherent fruit of China. On an industrial scale, peaches have also been growing in Pakistan, like in other states (China, Italy, and the United States) (Ali et al., 2014). Peach is in second placing among stone fruits that could be used fresh or processed (Bakht et al., 2012). It is commercially grown above sea level (1200–1500 m altitude) in a temperate climate with less rain (dry) during the winter and a modest summer worldwide (FAOSTAT, 2016). Based on total cultivated land for fruit production, peaches are grown on 19 % of land in Khyber Pakhtunkhwa, where in Mardan production is 3.64 thousand tons and in Malakand and Swat combined, 43.94 thousand tons (FAOSTAT, 2016). Dehydration of commodities for extended periods of time is the best way to minimize losses after picking, especially solar drying for quality maintenance (Selvi, 2020). Several process technologies (for example, canning, freezing, and dehydration) have been used to preserve food on an industrial level. Commodity dehydration procedures used by our forefathers are a good way to decrease loss rates due to their productivity in underdeveloped states (Abrol et al., 2014).

Ethylene and respiration speeds in peaches are very rapid on ripening due to their climacteric nature, which minimizes their life after picking and in storage (Alonso-Salinas et al., 2022). High water presence (fresh commodities) led to less market availability (Alonso-Salinas et al., 2022) and maximum wastage (FAO 2011). Due to its short postharvest life, its marketability is reduced, resulting in a significant cost and profit drop for the growers. Peach fruit production has received less attention due to its high perishability and limited postharvest life, which could be extended using several techniques for maximum availability (Pongener et al., 2011). At various growth levels, it is prone to certain diseases (such as scab, anthracnose, bacterial spot, brown rot, and vice versa) and pathogen attacks due to its high water content (Al-Hamdani et al., 2022). Being a perishable commodity (prone to post-harvest diseases), peaches transmit major issues during handling and shipping, leading to lower quality during storage (Wang et al., 2017).

Solar drying could be utilized to adequately maintain quality and extend shelf life for agricultural products. The presence of less than 10 % water in solar dried slices led to lower chemical changes, microbe damage, and rancidity. Solar dried products (fine texture with export quality) have been protected from dust and pathogens and exhibit high quality, resulting in uniform water loss along with minimal nutrient losses (Khattak et al., 2019). Solar energy is widely available in Pakistan. Solar collectors utilize solar energy to dehydrate commodities (García-Moreira et al., 2023), while solar radiation is helpful in wood drying too.

Solar drying is the best choice in areas receiving maximum solar irradiance in developing regions (García-Moreira et al., 2023). Peaches have a short shelf life due to their high moisture content, respiration rate, and ethylene production, which cause deterioration very quickly. This may also lead to serious economic losses for peach growers. The non-availability of proper storage, infrastructure, and packaging in Pakistan also affects the export of commodities. Considering the above issues, this study was carried out to study the influence of drying methods and slice thickness on the quality and shelf life of dried peaches.

## 2 Materials and methods

### 2.1 Experimental site and design

An experiment was laid out at Department of Agricultural Mechanization and Renewable Energy Technologies for the drying of peaches. Physio-chemical analyses were then carried out at the Department of Horticulture, The University of Agriculture Peshawar, Khyber Pakhtunkhwa, Pakistan, during the summer of 2022. It lies at 34.01° N latitude, 71.35° E longitude, 350 m above sea level, and is 1600 km north of the Indian Ocean (Alam et al., 2020). 5 °C is the minimum winter temperature and 45 °C is the average summer temperature (max rainfall = 78 mm in March, known as the wettest month), while June receives 7 mm of rainfall (Gilani et al., 2021). The experiment was repeated three times with three factors applied in a CRD (Completely Randomized Design).

### 2.2 Drying procedure of peaches

Peach cultivar “Indian Blood Red” was used in the experiment, which is self-fertile and has a round shape (Whealy & Thuente, 2001). High-quality peaches were used in the experiment; about 2 kg of peaches for each replication were cut into the desired slice thickness. The slice thickness was measured by a Vernier calliper, followed by initial water presence recording using the oven drying procedure. Perforated trays (80 %) were used for slice placement and then kept for drying, which continued up to 10 % water reduction (Santos et al., 2005). Similar procedures were carried out in the open sun (varying temperatures), while solar dying (60 °C) of peach slices was carried out in a flat plate solar collector fixed at Department of Agriculture Mechanization and Renewable Energy Technologies (Hanif, 2011). Similar procedures were carried out in the oven, where the temperature was also kept constant (about 60 °C). The dried peaches were stored in airtight boxes in a dark and cool place for up to 6 months, with the storage place temperature maintained at 60 °C (Abdullah, 2009). After drying, the fruit was stored for 30, 60, and 90 days at a relative humidity of 67–70 % in airtight boxes. The experiment was repeated three times with a three-factorial CRD (Completely Randomized Design).

### 2.3 Preparation of dried peaches juice

For the preparation of juice, 10 g of peach slices were taken in a juicer with 10 mL of distilled water to grind them properly.

### 2.4 Studied parameters

#### 2.4.1 Moisture content (%)

Slices were dried (<10 % moisture), while recording weight after every interval (using an electronic balance) using the below formula according to Karathanos (1999):

$$\mathrm{Mc}=Wi-\frac{\mathrm{Wf}}{\mathrm{Wi}}\times 100$$

Mc = moisture content, Wi = initial mass of the samples, Wf = final mass of the samples.

#### 2.4.2 Drying rate (g/g.cm^2^.h)

The loss of water per unit area of slices in per unit time represents drying rate, which was calculated utilizing the below formula (Hanif et al., 2012).

$$\mathrm{Dr}=\frac{(Wi-Wf}{\mathrm{Dm}\times Ap\times Dt}$$

Where Dr = drying rate, Wi = initial mass, Wf = final mass (g), Dm = dry matter (g), Ap = cross sectional area (cm^2^), and Dt = drying time (h).

#### 2.4.3 Drying time (hr)

The drying time was calculated by recording total number of hours taken by fruits to dry at required level.

#### 2.4.4 Total soluble solids (TSS) (^0^Brix)

The value of TSS (°Brix) was recorded through a refractometer by placing one drop of juice on a prism according to Fatima et al., (2023) method.

#### 2.4.5 Titratable acidity (TA) (%)

TA was calculated by taking 10 mL of juice, diluting it with distilled water in 100 mL beaker, and then titrating through 0.1 N NaOH; furthermore, for end point, 2–3 drops of phenolapthalein were added (Abidi et al., 2015).

#### 2.4.6 Total soluble solids (TSS) to acid ratio

TSS was divided by TA to get the ratio.

$$\mathrm{TSS}-acidratio=\frac{\mathrm{TotalSolubleSolids}}{\mathrm{Titratableacidity}}$$

#### 2.4.7 Ascorbic acid content (mg.100 g^−1^)

Dye method was utilized for ascorbic acid calculation (AOAC 1990).

$$\mathrm{Ascorbicacid}=\frac{T\times F\times 100}{D\times S}\times 100$$

Where F = standardization factor, T = dye (mL) used for sample- dye (mL) used for blank, D = sample for dilution (mL), S = diluted sample for titration (mL).

#### 2.4.8 Reducing sugars (%)

Reducing sugar content was calculated by the AOAC (2020). A 10 mL juice sample in a 100 mL volumetric flask was taken, and distilled water (90 mL) was used for volume increase. 5 mL of Fehling A, Fehling B solutions, along with 10 mL of distilled water, were added to a conical flask. After that, add the sample solution to the boiling solution from the burette, and for the end point (red brick color shown), 2–3 drops of Methylene Blue were added to the boiling solution.

#### 2.4.9 Non-reducing sugars (%)

Non-reducing sugars were calculated by AOAC (2012). In a volumetric flask (100 mL), 10 mL of juice was mixed with 90 mL of distilled water. 10 mL of distilled water and 5 mL of Fehling A, Fehling B solutions were added to conical flask. 10 mL of each HCl and NaOH were taken in a flask by adding 250 mL of distilled water to raise the volume. After that, add the sample solution to the boiling solution from the burette, and to obtain the end point, add 2–3 drops of methylene blue to the boiling solution.

#### 2.4.10 Disease incidence (%)

Disease percentage was measured according to Fatima et al., (2023) method through the following formula with regular disease symptoms checking:

$$Diseaseincidence\left(\%\right)=\frac{\mathrm{Weightofinfectedsample}}{\mathrm{Totalweightofsample}}\times 100$$

### 2.5 Organoleptic attributes

#### 2.5.1 Color score

For color scoring, the Hedonic scale (Larmond, 1987) was used, where randomly selected dried samples were presented before a group of food specialists for scoring (0–10).

#### 2.5.2 Taste score

Taste scoring was done by 20 individuals at the department of food science using the hedonic scale defined by Lawless and Heyman (2010), where judges rated the dried slices among 9 points as a scale for the study.

#### 2.5.3 Sample preparation for physiochemical studies

To obtain samples with representative chemical components for specific drying settings, dried peach slices were crushed into powder and put through a 60-mesh sieve. A centrifuge tube was filled with a sample of peach slice powder (2.0 g), 20 mL of 50 % ethanol (v/v), and the liquid was mixed for 2 min using a vortex mixer (VORTEX-5). Sample residue was extracted twice with 20 mL of 50 % ethanol (v/v) following centrifugation at 5000 rpm for 10 min. The supernatant was then collected. With 50 % ethanol (v/v), the mixed supernatants were brought to volume of 100 mL. Until further bioactive compound investigation, the extracts were kept at 4 °C (Mukhtar et al., 2022).

### 2.6 Determination of total phenolic contents (TPC; mg GAE/g)

Folin-Ciocalteu reagent was used to determine phenolic content (Emilio et al., 2011). After mixing a 20 µL sample with 100 µL of Folin-Ciocalteu reagent, the mixture was incubated at 37 °C for 60 s before 80 µL of 7.5 % (w/v) sodium bicarbonate solution was added. An absorbance reading at 765 nm was taken after the samples were once again combined and incubated at 37 °C for 15 min. Gallic acid calibration curve was used to evaluate TPC, and results were displayed as mg of gallic acid equivalents (GAE) per 100 g of dry weight (DW).

### 2.7 Determination of total flavonoid content (TFC; mg RE/g)

With slight adjustments, the aluminium chloride colorimetric method was utilized to estimate total flavonoid concentration (Souza et al., 2014). In a 10 mL tube, sample extract (1 mL) was combined with 0.5 mL of 50 % NaNO_2_ solution (w/v), 4 mL of 50 % ethanol (v/v), and 1 mL of sample. After the reaction had been going for 6 min, the tube received 0.5 mL of 10 % AlCl_3_ solution (w/v). 4 mL of 1 M NaOH were added after 6 min. After completely blending, the aforementioned solution was set aside for 10 min. At 510 nm, the absorbance was measured. Rutin was used in various quantities (0–500 mg/L), and a calibration curve was produced. The amount of rutin equivalent (RE) in one gramme of dry weight (mg RE/g) was used to indicate TFC.

### 2.8 Determination of antioxidant activity (AA; mg TE/g)

Using the products' 2,2-diphenyl-1-picrylhydrazyl (DPPH) radical scavenging activity assay, antioxidants in the samples before and after drying were identified according to Fatima et al., (2023) method. The reaction was place for two hours at 25 °C with 1.5 mL of DPHH added to various aliquots of ethanol. With the use of spectrometer (Model: Hitachi UK-1900, UK), the absorbance spectrum was determined at 500 nm. We calculated sample mass required to block 50 % DPPH. The calculations were made using the equation mentioned by Delgado et al. (2016) and expressed as the percentage of antioxidant retention.

$$Antioxidants\;Retention\;\left(\%\right)=\;100-\;\frac{\left(The\;initial\;antioxidant\;retention\;capacity-The\;final\;antioxidant\;retention\;capacity\;\right)}{The\;initial\;antioxidant\;retention\;capacity}\times 100$$

### 2.9 Statistical analysis

ANOVA (analysis of variance) was laid out to detect differences as well as interactions for the mentioned parameters using the statistical software Statistix 8.1, and Duncan multiple range test was done where differences were significant (Jan et al., 2009).

## 3 Results

### 3.1 Moisture content (%)

**Fig. 1:**
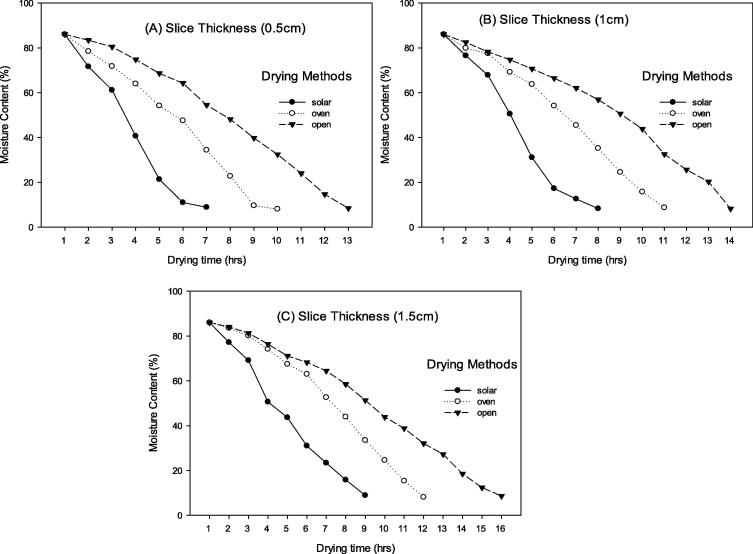
Moisture content (%) of peach slices of (A) 0.5 cm (B) 1.0 cm and (C) 1.5 cm thickness dried through three different drying methods**.**

The maximum moisture content reduction (8.9 %) in 0.5 cm slice thickness was observed in solar collector drying by taking 7 h, followed by oven drying (8.1 %) by taking 10 h, and the minimum moisture content reduction (8.45 %) was recorded in open sun drying by having 13 h of drying duration. Similarly, the maximum moisture content reduction (8.3 %) in 1.0 cm slice thickness of peaches was observed in solar collector drying by taking 8 h, followed by oven drying (8.75 %) by taking 11 h, and the minimum moisture content reduction (8.25 %) was recorded in open sun drying with 14 h of drying duration. Furthermore, in 1.5 cm slice thickness, the maximum moisture content reduction (8.9 %) was observed in solar collector drying by taking 9 h, followed by oven drying (8.1 %) by taking 12 h, and the minimum moisture content reduction (8.6 %) was recorded in open sun drying by having 16 h of drying duration (Fig. 1).

### 3.2 Drying rate (g/g.cm^2^.hr)

**Fig. 2:**
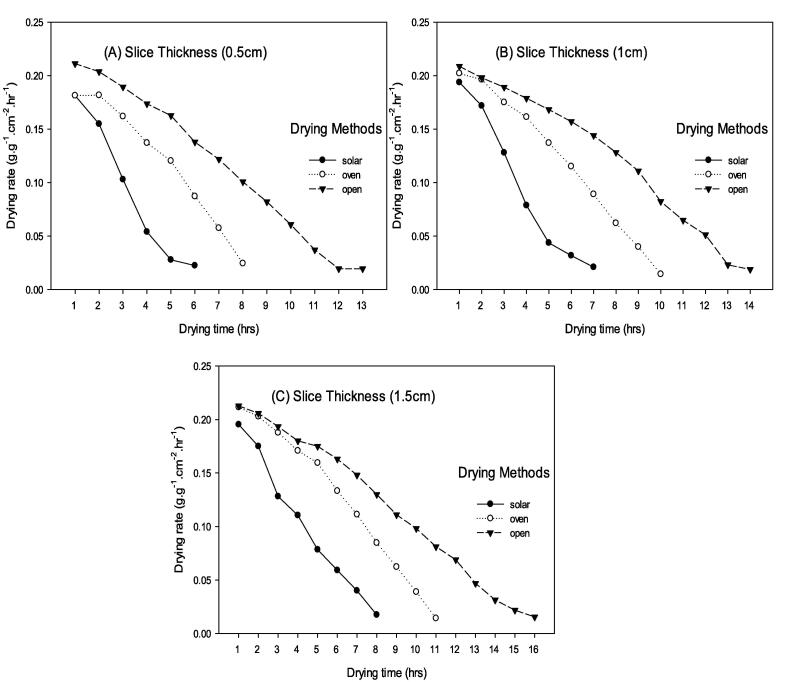
Drying rate (g/g.cm^2^.hr) of peach slices of (A) 0.5 cm (B) 1.0 cm and (C) 1.5 cm thickness dried through three different drying methods.

The maximum drying rate (0.023 g/g.cm^2^.hr) in 0.5 cm sliced peaches was observed in solar collector drying, which took 7 h, followed by oven drying (0.025 g/g.cm^2^.hr) which took 10 h. However, the minimum drying rate (0.020 g/g.cm^2^.hr) was recorded in open sun drying with 13 h of drying duration. Similarly, the maximum drying rate (0.018 g/g.cm^2^.hr) in 1.0 cm sliced peaches was observed in solar collector drying by taking 8 h, followed by oven drying (0.014 g/g.cm^2^.hr) by taking 11 h, and the minimum drying rate (0.023 g/g.cm^2^.hr) was recorded in open sun drying having 14 h of drying duration. The maximum drying rate (0.017 g/g.cm^2^.hr) in 1.5 cm sliced peaches was observed in solar collector drying by taking 9 h, followed by oven drying (0.009 g/g.cm^2^.hr) by taking 12 h, and the minimum drying rate (0.006 g/g.cm^2^.hr) in open sun drying having 16 h of drying duration (Fig. 2).

### 3.3 Drying time (hr)

**Fig. 3:**
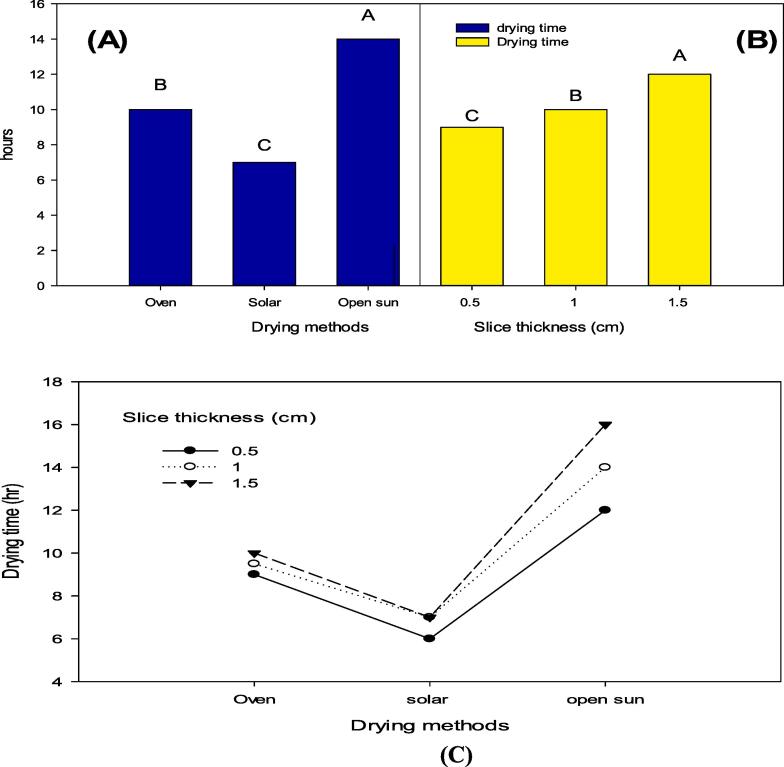
Drying time (hr) of peach slices as affected by (A) drying methods, (B) slice thickness and interaction of drying methods and slice thickness. Means followed by the same letters (A-C) in their respective column do not differ significantly according to Duncan multiple range test at P ≤ 0.01.

Drying methods represented that the maximum (13 hrs) drying time was recorded in peaches dried by open sun method, followed by oven drying (10 hrs), while the lowest drying time (7 hrs) was observed in peaches dried by the solar drying method with a 0.5 cm slice thickness (Fig. 3 A). Slice thickness has also considerably affected the drying time of dried peaches. Slice thickness of 1.5 cm shows maximum drying time (16 hrs), followed by 1.0 cm having drying time (10 hrs), while minimum drying time (9 hrs) was observed in slice thickness of 0.5 cm (Fig. 3 B). The interactions of DM × ST had significant effects on drying time of dried peaches, where the maximum drying time (16 hrs) was observed in slices with thickness of 1.5 cm dried by open sun drying and the lowest (7 hrs) was observed in slices with thickness of 0.5 cm dried by solar drying (Fig. 3 C).

### 3.4 Total soluble solids (Brix°)

Drying methods (DM), slice thickness (ST) and storage duration (SD) have significantly affected the TSS of dried peaches. All the 2-way interactions (DM × ST, DM × SD and ST × SD) were significant, while the 3 way interactions (DM × ST × SD) had non-significant effect on TSS of dried peaches. Drying methods showed that the maximum TSS (8.18 °Brix) was recorded for solar drying, followed by TSS (7.43 °Brix) in oven drying. The lowest TSS (6.95 °Brix) was recorded in open, sun-dried slices. Similarly, the maximum TSS (8.03 °Brix) was observed in peach fruits with a 1.5 cm slice thickness. The lowest TSS (7.03 °Brix) was taken from dried peaches with 0.5 cm slice thickness, which is not significantly different from TSS (7.51 °Brix) in 1.0 cm sliced peaches. Storage durations also had significant effect on the TSS of dried peaches. An increase in TSS in dried peaches was observed during storage. Total soluble solids in freshly harvested peaches were 11.70 °Brix. However, a TSS of 6.95, 8.59, and 8.90 °Brix was observed in peaches after drying for 30, 60, and 90 days, respectively (Table 1). The interaction of drying methods and storage duration showed that the highest TSS (10.03 °Brix) was recorded in peaches dried through open sun drying after 90 days of storage, while lowest TSS (5.33 °Brix) was observed in open sun-dried samples during 60 days of storage. Similarly, interaction of drying methods and slice thickness showed that maximum TSS (9.19 °Brix) was observed in slice thickness of 1.5 cm dried by open sun drying, while lowest (7.21 °Brix) was recorded in slice thickness of 1.0 cm dried by oven drying. Furthermore, the interaction of storage duration and slice thickness showed the highest TSS (10.26 °Brix) during 90 days of storage with a 1.5 cm slice thickness, while the lowest TSS (5.57 cm) was recorded with a 1.5 cm slice thickness at 60 days of storage.

**Table 1:** Total soluble solids (TSS Brix^0^), titratable acidity (%) and TSS to acid ratio and disease incidence of dried peach slices as affected by drying methods, slice thickness and storage duration.

Treatment	Parameters		
Drying methods (DM)	TSS (Brix^0^)	TA (%)	TSS-Acid ratio
Oven	7.43^b^	6.18^b^	1.20^b^
Solar	8.18^a^	7.74^a^	1.05^c^
Open	6.95^c^	4.19^c^	1.66^a^
*LSD values*	0.46	1.28	0.13
Slice Thickness (ST; cm)			
0.5	7.03^b^	2.66^c^	2.64^a^
1.0	7.51^b^	5.85^b^	1.29^b^
1.5	8.03^a^	9.60^a^	0.84^c^
*LSD value*	0.48	1.28	0.13
Storage durations (SD) (Days)			
Before drying	11.70	0.308	37.98
Fresh (After drying)	5.696^c^	15.54^a^	0.37^d^
30	6.956^b^	4.034^b^	1.72^c^
60	8.59^a^	2.59^bc^	3.32^b^
90	8.904^a^	1.97^c^	4.52^a^
*LSD values*	0.56	1.47	0.56
Interaction			
DM × ST	**	**	NS
DM × SD	**	**	**
ST × SD	**	**	NS
DM × ST × SD	NS	NS	NS

Means followed by the same letters (a, b, c, and, d) in their respective column do not differ significantly according to Duncan multiple range test at P ≤ 0.01.

NS = Non-significant and **= Significant at p ≤ 0.01.

### 3.5 Titratable acidity (%)

Drying methods showed that the maximum (7.74 %) TA was recorded in peaches dried by the solar method, followed by oven drying (6.18 %), while the lowest TA (4.19 %) was observed in peaches dried by open sun drying method. Similarly, slices with a thickness of 1.5 cm showed a higher TA (9.60 %), while the lowest TA (2.66 %) was observed in slices with a thickness of 0.5 cm. Titratable acidity increased with increase in slice thickness, and storage duration of dried peaches for TA showed that TA decreased as storage duration extended. In the first 30 days, 4.03 % TA was seen, followed by 60 days with TA (2.59 %), and the lowest TA was seen in 90 days (1.97 %). The fresh TA was 0.31 %, which increased to 5.54 % after drying (Table 1).

The interaction of drying methods and storage duration showed that the highest TA (18.51 %) was recorded in peaches dried through open sun drying in storage for 30 days, while the lowest TA (0.44 %) was observed in solar dried samples during 90 days of storage. The interaction of drying methods and slice thickness showed that maximum TA (9.75 %) was observed in slice thickness of 1.5 cm dried by open sun drying and lowest (1.23 %) was observed in slice thickness of 0.5 cm dried by solar drying. The interaction of storage duration and slice thickness showed the highest TA (6.37 %) during 30 days of storage with a 1.5 cm slice thickness, while the lowest TA (1.10 %) was recorded with a 0.5 cm slice thickness at 90 days of storage. The interaction of slice thickness and storage duration showed that the highest TA (%) was recorded in peaches dried through open sun drying in storage for 30 days, while the lowest TA (%) was observed in solar dried samples during 90 days of storage.

### 3.6 TSS to acid ratio

Drying methods, slice thickness, storage conditions, and the interaction of DM × SD had a considerable effect on the TSS-acid ratio of dried peaches, while all other interactions were non-significant. The drying methods of peaches indicate that a higher ratio (1.66) was found in open solar-dried peaches, followed by oven-dried peaches (1.20), while the lowest ratio (1.05) was recorded in solar-dried peaches. Similarly, slice thickness (0.5 cm) has a higher ratio (2.64), followed by 1.0 cm and 1.5 cm slice thickness (1.29 and 0.84), respectively. Storage duration showed that the ratio becomes enhanced with storage duration. The ratio in fresh harvested samples was 37.98, which decreased to 0.37 after drying. After a duration of 30 days, the ratio was 1.72, followed by 60 days (3.32), and the highest ratio was observed in 90 days (4.52) (Table 1). The interaction of drying methods or storage duration showed that the highest TSS-Acid ratio (14.23) was recorded in peaches dried through solar drying in storage for 90 days, while the lowest TSS-Acid ratio (1.56) was observed in oven-dried samples during 30 days of storage.

### 3.7 Ascorbic acid content (mg.100 g^−1^)

Ascorbic acid content was enhanced by increasing slice thickness. 1.5 cm slice thickness showed more ascorbic acid content (19.85 mg.100 g^−1^) followed by 21.61 mg.100 g^−1^ in 1.0 cm slice thickness, while lower ascorbic acid content (24.59 mg.100 g^−1^) was recorded in 0.5 cm slice thickness. Similarly, maximum ascorbic acid content (23.18 mg.100 g^−1^) was seen in solar dried samples, followed by oven-dried samples (21.74 mg.100 g^−1^) and the lowest ascorbic acid content (21.13 mg.100 g^−1^) was observed in open-sun-dried peaches. The data taken during storage durations of 90 days showed that ascorbic acid content increased with lengthening the time. The fresh harvested samples had 8.13 mg.100 g^−1^ ascorbic acid, and 14.1 mg.100 g^−1^ was observed after drying. The 30 days of storage showed 20.3 mg.100 g^−1^ ascorbic acid content, followed by 25.63 mg.100 g^−1^ after 60 days, and the lowest ascorbic acid content (28.09 mg.100 g^−1^) was noted in the 90 days of storage (Table 2).

**Table 2:** Ascorbic acid (mg.100 g^−1^), Reducing sugars (%) and Non-reducing sugars (%) and disease incidence (%) of dried peach slices as affected by drying methods, slice thickness and storage duration.

Treatment	Parameters		
Drying methods (DM)	Ascorbic acid	Non-Reducing sugar	Reducing sugar
Oven	21.74^b^	71.83^b^	96.13^b^
Solar	23.18^a^	69.11^b^	103.03^a^
Open	21.13^b^	96.03^a^	81.79^c^
*LSD values*	1.44	10.83	3.33
Slice Thickness (ST;cm)			
0.5	19.85^c^	77.72^c^	88.87^b^
1.0	21.61^b^	78.73^b^	95.09^a^
1.5	24.59^a^	80.52^a^	96.29^a^
*LSD value*	1.44	10.83	3.33
Storage durations (Days)			
Before drying	8.130	74.81	75.07
Fresh (After drying)	28.093^a^	173.07^a^	80.64^d^
30	25.63^b^	54.63^c^	89.31^c^
60	20.30^c^	47.78 ^cd^	100.00^b^
90	14.04^d^	40.48 ^d^	110.63^a^
*LSD values*	1.67	12.51	3.84
Interaction			
DM × ST	**	NS	**
DM × SD	**	NS	**
ST × SD	**	NS	NS
DM × ST × SD	NS	NS	NS

Means followed by the same letters (a, b, c, and, d) in their respective column do not differ significantly according to Duncan multiple range test at P ≤ 0.01.

NS = Non-significant and **= Significant at p ≤ 0.01.

The interaction of drying methods and storage duration revealed that the highest ascorbic acid content (29.73 mg.100 g^−1^) was recorded in peaches dried through solar drying in storage for 60 days, while the lowest ascorbic acid (17.6 mg.100 g^−1^) was detected in open sun-dried samples during 90 days of storage. While the interaction of drying methods and slice thickness revealed that maximum ascorbic acid (22.57 mg.100 g^−1^) was detected in slices with thickness of 1.5 cm dried by solar drying. While the lowest ascorbic acid (15.88 mg.100 g^−1^) was observed in 0.5 cm slice thickness dried by open sun drying. Furthermore, interaction of storage duration and slice thickness showed the highest ascorbic acid (31.03 mg.100 g^−1^) during 90 days of storage having a 1.5 cm slice thickness, while the lowest ascorbic acid (16.98 mg.100 g^−1^) was recorded with a 0.5 cm slice thickness at 30 days of storage.

### 3.8 Non-reducing sugars (%)

The highest non-reducing sugar (96.03 %) was seen in samples of open sun drying, followed by oven drying (71.83 %), while the lowest (69.11 %) was found in solar dried samples. In terms of storage time, dried samples had 173.07 % non-reducing sugar after drying, which became less (54.63 %) after 30 days and reached 40.48 % in 90 days. The freshly picked samples had 74.81 % reducing sugars. Slice thickness has no significant effect on non-reducing sugars. Non-reducing sugars were found at 77.72, 78.73, and 80.52 % in 0.5, 1.0, and 1.5 cm slice thickness dried samples (Table 2). All the two- and three-way interactions were non-significant.

### 3.9 Reducing sugars (%)

Storage time shows that reducing sugar for freshly picked samples was 75.07 %. After drying, it became 80.64 %. The increase in reducing sugars was seen during 90 days of storage. In 30 days, reducing sugar was (89.31 %), followed by 100.63 %, and finally the highest (110.00 %) was recorded in 90 days. In the case of drying methods, the highest (103.03 %) reducing sugar was recorded in solar dried samples, followed by oven dried (96.13 %), while the lowest (81.79 %) was recorded in open-sun-dried peaches. More reducing sugar (96.29 %) was observed in 1.5 cm slice thickness, followed by (95.09 %) in 1.0 cm, which had no statistical difference between them. The lowest (88.87 %) was observed at 0.5 cm slice thickness (Table 2). whereas 2-way interaction has significant effects except slice thickness and storage duration. The interaction of drying methods and storage duration showed that the highest reducing sugar (115.89 %) was recorded in peaches dried through open sun drying in storage for 90 days, while the lowest reducing sugar (79.71 %) was observed in solar-dried samples during 30 days of storage. The interaction of drying methods and slice thickness showed that maximum reducing sugar (106.93 %) was observed in slices with thickness of 1.0 cm dried by open sun drying. while the lowest reducing sugar (75.09 %) was observed in 0.5 cm slice thickness dried by solar drying.

### 3.10 Disease incidence (%)

In the case of drying methods, the highest (14.00 %) disease attack was observed in samples of open sun drying, followed by oven drying (8.08 %), and the minimum (3.0 %) disease attack was recorded in open sun drying. Regarding slice thickness, the maximum disease incidence (11.50 %) was observed in dried peaches with a 1.5 cm slice thickness. The lowest disease incidence (5.14 %) was detected for dried peaches with a 0.5 cm slice thickness. Storage durations also had significant effect on the disease incidence of dried peaches. The maximum disease incidence (12.00 %) and lowest (10.93 %) were noticed at 30 and 90-day intervals (Table 3). The interaction of drying methods and storage duration showed that the highest disease incidence (19.33 %) was recorded in peaches dried through open sun drying in storage for 90 days, while the lowest disease incidence (10.67 %) was observed in solar-dried samples during 30 days of storage. Similarly, the interaction of drying methods and slice thickness showed that the maximum disease incidence (17.83 %) was observed in slices with thickness of 1.5 cm dried by open sun drying. While the lowest disease incidence (5.89 %) was observed in slices of 0.5 cm thickness dried by solar drying, Furthermore, the interaction of storage duration and slice thickness showed the highest disease incidence (16.44 %) during 30 days of storage with a 1.5 cm slice thickness, while the lowest disease incidence (5.89 %) was recorded with a 0.5 cm slice thickness at 30 days of storage.

**Table 3:** Disease incidence (%), taste score and color score of dried peach slices as affected by drying methods, slice thickness and storage duration.

Treatment	Parameters		
Drying methods (DM)	Disease incidence	Taste score	Color score
Oven	8.08^b^	7.08^b^	7.33^b^
Solar	3.00^c^	8.79^a^	7.97^a^
Open	14.00^a^	6.67^b^	6.94^c^
*LSD values*	3.63	0.51	0.35
Slice Thickness (ST;cm)			
0.5	5.44^c^	8.06^a^	8.03^a^
1.0	8.44^b^	7.40^b^	7.52^b^
1.5	11.50^a^	7.08^b^	6.69^c^
*LSD value*	3.63	0.51	0.35
Storage durations (Days)			
Before drying	0.00	7.40	7.02
Fresh (After drying)	0.00^c^	8.02^a^	7.45^a^
30	12.00^a^	7.61^ab^	7.44^a^
60	10.93^b^	7.36^b^	7.40^a^
90	10.52^b^	7.04^b^	7.34^a^
*LSD values*	4.19	0.59	NS
Interaction			
DM × ST	**	**	**
DM × SD	**	NS	NS
ST × SD	**	NS	NS
DM × ST × SD	NS	NS	NS

Means followed by the same letters (a, b, c, and, d) in their respective column do not differ significantly according to Duncan multiple range test at P ≤ 0.01.

NS = Non-significant and **= Significant at p ≤ 0.01.

### 3.11 Taste score

All the 2-way interactions (DM × SD and ST × SD) and 3-way interactions (DM × ST × SD) were found to be non-significant except DM × ST on the flavor of dried peaches. Drying methods showed that the maximum taste score (8.79) was recorded in solar drying, followed by 7.08 in oven-dried samples, while the lowest (6.67) was recorded in open sun-dried peaches. Regarding slice thickness, the maximum score (8.06) was given to peach fruits with a 0.5 cm slice thickness. The lowest score (7.08) was observed for dried peaches with a 1.5-cm slice thickness. Storage durations also had significant effect on the flavor of dried peaches. The maximum mean score value for taste (7.61) and lowest mean score value (7.04) were noticed at 30 and 90-day intervals, and the flavor became poor (Table 3). The interaction of drying methods and slice thickness showed that maximum taste score (9.08) was observed in slices with a thickness of 0.5 cm dried by solar drying, while slices of 1.5 cm thickness dried in the open sun received the lowest taste score (5.88).

### 3.12 Color score

Drying methods showed that the maximum color score (7.97) was given to solar drying, followed by 7.33 for oven drying. The lowest color score (6.94) was given to peach fruits dried in the open sun. Regarding slice thickness, the maximum color score (8.03) was observed in peach fruits with a 0.5 cm slice thickness. The lowest color score (6.69) was observed for dried peaches with a 1.5 cm slice thickness. Storage durations also had significant effect on the color of dried peaches. The sensory scores of color reduced considerably in all samples during 90 days of storage, but scores were found to be within acceptable limits. At 30 days of storage, the maximum score of 7.44 was observed, followed by 7.40 at 60 days. After 90 days of storage, scores decreased to 7.34. The results indicate that various types of drying methods significantly affected color of all samples, and judges highly preferred the color of peaches dried by solar drying with a 0.5 cm slice thickness (Table 3). The interaction of drying methods and slice thickness showed that maximum color score (8.25) was observed in slices with a thickness of 0.5 cm dried by solar drying, while the lowest color score (5.90) was observed in slices of 1.5 cm thickness dried by solar drying.

### 3.13 Total phenolic content (mg GAE/g)

Drying methods showed that maximum total phenols (14.77 mg GAE/g) were observed in solar drying, followed by 12.57 mg GAE/g in oven drying. The lowest TPC (11.07 mg GAE/g) was recorded in peach slices dried in the open sun. Regarding slice thickness, the maximum TPC (13.51 mg GAE/g) was observed in peach slices with a thickness of 1.5 cm. The lowest TPC (12.84 mg GAE/g) was observed for dried peaches with a 0.5 cm slice thickness. Storage durations also had significant effect on the TPC of dried peaches. At 30 days of storage, the maximum TPC (13.34 mg GAE/g) was observed, followed by 13.19 mg GAE/g at 60 days. After 90 days of the storage period, TPC decreased to 12.53 mg GAE/g. The results indicate that various types of drying methods, slice thickness, and storage duration had significant effect on the TPC of all samples (Table 4).

**Table 4:** Total phenolic content (mg GAE/g), Total flavonoids content (mg RE/g), and antioxidant activity of dried peach slices as affected by drying methods, slice thickness and storage duration**.**

Treatment	Parameters		
Drying methods (DM)	Total phenolic	Total flavonoids	Antioxidants
Oven	12.57^b^	15.98^b^	4.98^b^
Solar	14.77^a^	18.13^a^	4.48^c^
Open	11.07^c^	11.56^c^	7.18^a^
*LSD values*	0.158	0.146	0.164
Slice Thickness (ST;cm)			
0.5	12.84^b^	14.76^b^	5.75^a^
1.0	13.30^a^	15.38^a^	5.53^b^
1.5	13.51^a^	15.44^a^	5.37^c^
*LSD value*	0.158	0.146	0.164
Storage durations (Days)			
Before drying	25.56	27.84	10.90
Fresh (After drying)	13.81^a^	15.65^a^	5.95^a^
30	13.34^a^	15.30^a^	5.68^a^
60	13.19^a^	15.19^a^	5.52^a^
90	12.53^b^	14.63^b^	5.04^b^
*LSD values*	0.182	0.169	0.189
Interaction			
DM × ST	**	NS	**
DM × SD	**	NS	NS
ST × SD	**	**	**
DM × ST × SD	NS	NS	NS

Means followed by the same letters (a, b, c, and, d) in their respective column do not differ significantly according to Duncan multiple range test at P ≤ 0.01.

NS = Non-significant, and **= Significant at p ≤ 0.01.

### 3.14 Total flavonoids content (mg RE/g)

Storage time shows that the TFC for freshly picked samples was 27.84 mg RE/g. After drying, it became 15.65 mg RE/g. The decrease in TFC was seen during 90 days of storage. In 90 days, TFC was 14.63 mg RE/g) followed by 15.19 mg RE/g, and finally the highest (15.30 mg RE/g) was recorded in 30 days. In the case of drying methods, the highest (18.13 mg RE/g) TFC was found in solar dried samples, followed by oven dried (15.98 mg RE/g), while the lowest (11.56 mg RE/g) was recorded in open-sun-dried peaches. More TFC (15.44 mg RE/g) was observed in 1.5 cm slice thickness, followed by (15.38 mg RE/g) in 1.0 cm, which has no statistical difference between them. The lowest (14.76 mg RE/g) was observed at 0.5 cm slice thickness (Table 4), whereas 2-way interactions of only slice thickness and storage duration had significant effects among other 2-way interactions.

### 3.15 Antioxidant activity (mg TE/g)

Drying methods showed that the maximum (7.18 mg TE/g) AA was recorded in peaches dried by the open sun method, followed by oven drying (4.98 mg TE/g), while the lowest AA (4.48 mg TE/g) was observed in peaches dried by the solar drying method. Similarly, slices with a thickness of 0.5 cm showed a higher AA (5.75 mg TE/g), while the lowest AA (5.37 mg TE/g) was observed in slices with a thickness of 1.5 cm. Antioxidant activity decreased with an increase in slice thickness and storage duration of dried peaches for AA. At first, 5.68 mg TE/g AA was seen at 30 days, followed by 60 days of AA (5.52 mg TE/g), and the lowest AA was seen at 90 days (5.04 mg TE/g). The fresh AA was 10.90 mg TE/g, which decreased about 5.95 mg TE/g after drying (Table 4), whereas the 2-way interaction of DM × ST and ST × SD had significant effects.

## 4 Discussion

Initially, more energy is absorbed by water due to the high water presence on the slice surface, which speeds up the drying rate but, after some time, becomes slow due to heat penetration through the dried layer (Sharma et al., 2005, Ullah et al., 2022). Loss of high moisture content from sample surface led to lower drying rate (Togrul and Pehlivan, 2002) because water movement in slices came to the surface and evaporated to the atmosphere from the surface (Akpinar et al., 2003). Highly perishable commodities have a lower drying rate due to more moisture diffusion within slices as compared to moisture loss from the surface to the air. With longer drying times, the samples' moisture content dropped. The rate of moisture evaporation is highest at the beginning of drying cycle and gradually declines as drying progresses (Hussain et al., 1972).

When horticulture products are dried in an oven, the polar water molecules interact with the heat of the oven, causing a higher moisture loss than when dried in the open sun (Schiffmann, 1992, El-Beltagi et al., 2022a). The drying method utilizing the open sun had the highest value for the moisture content of dry slices, followed by the oven. Due to the high temperature and equal distribution of heat during oven drying, the drying time of the water was shortened (Winangsih et al., 2013). Results were recorded similar to those of Ghasemi et al. (2013), showing that flat plate solar drying was better as compared to other methods. During the falling rate period, the moisture transfer was governed by diffusion. Uniform and consistent moisture loss in solar collectors maintains quality (Hanif et al., 2013). Moisture in drying materials can quickly and directly absorb microwave energy, which causes the substance to heat up (Puente-Díaz et al., 2013). The rapid temperature increase caused by the high vapor pressure that develops inside the product as a result of the microwaves' high heating rate (Nahimana and Zhang, 2011) causes water to transfer to the product's surface very quickly. Additionally, Bajoub et al. (2023) claimed that oven drying was superior to open-air sun drying. Sun drying takes longer to complete and is influenced by environmental factors including relative humidity and ambient air temperature (Rajkumar, 2007).

Water diffuses more quickly from thinner slices than from whole (thicker) slices due to the more exposed surface to the heating medium (Sugito et al., 2013). The outcomes are very similar to Kabiru et al. (2013), who reported that increasing slice thickness increased the moisture content and also took more time to dry. The dried substance dries more quickly the thinner it is. Temperature and packaging materials have a significant impact on how much water is lost during storage (Gonzalo et al., 2014), although drying techniques can affect the water status of stored samples (Suna et al., 2014).

Loss of moisture from fruit was very consistent with drying rate of 0.45 g/g.cm^2^.hr, which declined after 10 h. The loss of moisture and drying rate results are similar to the results of Thao et al., (2023). A high drying rate occurred initially for up to 6 h due to availability of free water molecules (An et al., 2023), but later on drying rate became slower. Compared to thicker samples of agricultural commodities, it was found that samples that were thinly sliced dried more quickly. Other horticultural items showed similar outcomes as well (Loha et al., 2012). The dense nature of peaches led to a lower drying rate (Anbu et al., 2016). The drying rate of solar drying is faster than that of natural sun drying, which requires a longer time. Fruits dried by solar drying are characterized by low-cost and energy savings as compared to oven drying (Skåra et al., 2022).

Drying time increases with an increase in slice thickness, as observed. Higher temperatures and less humidity in the oven lead to a shorter drying period and a higher drying rate of products, resulting in maximum water loss in less time (Aly et al., 2012, Akladious and Mohamed, 2017). The tendency of our results is strongly supported by those acquired by Khater (2020) and Khater and Bahnasawy (2017). During the summer months, where solar radiation is intense and ambient humidity is low, the most important factor is the higher temperature of the collector, which leads to faster drying with less drying time (Marwati, 2017). Aggarwal et al. (2010) dried various horticultural products in open sun, oven, and solar dryer to check nutritional quality, which was superior in the solar collector to open sun-dried products. The texture becomes rough with more drying time and thicker slices (Mengisst and Emire, 2022) as more time is wasted waiting for water molecules to come to the surface (Kongdej, 2011). Similarly, slices thicker in size dried in the open sun have a longer drying time than those dried in the solar sun, and thinner slice thickness have a lower drying time than those dried in the solar sun, but a faster drying rate because of more area exposure and lower distance travel for heat in the product (Ahmad et al., 2013).

TSS is one of the qualitative factors that consist of minerals, vitamins, etc. but not water (Baldwin, 1994). With the passage of time, TSS increased to some extent (conversion of starches to glucose and fructose) (Wills et al., 1980). Starches are sweeter than glucose and fructose, which is why fruit becomes sweeter as TSS increases (Khan et al., 2020). An increase in TSS occurs as moisture content reduces during drying, which leads to solid dilution. Rather than fresh samples, an increase in TSS occurs due to a reduction of moisture below 10 % in dried peaches. Suna et al., (2014) recorded a significant correlation between dry matter and mineral elements during drying, and the results are in strong correlation with our findings. Total soluble solids increased with increased temperature because of carbohydrate biosynthetic enzyme activity variations and transpiration also increased (Gupta et al., 2023).

During storage conditions, Hermandz-Munoz et al., (2008) recorded that TSS was observed to be enhanced due to respiration and metabolic reactions that covert disaccharides to monosaccharides, prospecting to pectin, and sucrose to fructose or glucose (Sharma et al., 2013, El-Beltagi et al., 2023a) reported similar records that TSS increased due to respiration, which enhanced metabolite synthesis leads to carbohydrates conversion to sugars and furthermore soluble compounds made due to pectin, hemicellulose, and cellulose degradation (Echeverria and Ismail, 1990). Moreover, TSS decline occurs with declines in respiration rates (Yaman and Bayoindirli, 2002).

TSS is the combination of water and total solids of fruit, and it is strongly linked with reducing sugars (fructose and glucose). Destriyani et al., (2014) reported a considerable influence of slice thickness on TSS, TSS increased with water loss, and thinner slice thickness dehydrated quickly. Beside initial moisture, thinner slices have the best quality due to less exposure to heat, which could be observed by comparing solar-dried samples to open-sun-dried samples for TSS (Evrendilek, 2016). Keeping certain factors (design, structure, and dryer) in mind led to proper drying method selection for TSS-specific commodities (Sneha and Prahlad, 2018). Our results are in accordance with Bhat et al., (2014), who recorded the maximum TSS of dried pomegranate.

TA in dried peaches declined during storage conditions due to acid utilization for reducing sugars conversion into non-reducing sugars. This conversion might occur because of the chemical interaction of organic constituents found in fruit as a result of enzyme activity and storage temperature (Kaushik, 1997). Respiration and TA have an inverse relationship with each other, and a reduction in TA is due to higher respiration (Ghafir et al., 2009). Gupta (2007) also reported a reduced trend of titratable acidity during osmosis, which may have occurred due to acid leaching. During storage, TA decline occurs due to degradation of malic and citric acids as a result of respiration, while acids remain constant in fruits with lower respiration rather than respired fruits in storage conditions (Baritelle et al., 2001). In osmo dried fruit, Kumar (2013) reported a decline in titratable acidity and the highest acidity in open sun-dried fruits (Gallali et al., 2000). Evrendilek (2016) also recorded more TA in open sun-dried samples due to the longer drying time, which results in acid accumulation as a result of carbohydrates degradation (Sneha and Prahlad, 2018).

For consumer acceptability, the fruits should have a higher TSS-Acid ratio (Sturm et al., 2003), and there is a lower TSS-Acid ratio in freshly harvested fruits, which becomes higher during storage as a result of acids conversion to sugars (Shahid and Abbasi, 2011). Workneh et al., (2012) recorded maximum values for certain parameters (moisture loss, TSS, vitamin C, and TSS-acid ratio) with lower TA and drying time in solar-dried slices than in oven dried samples. Based on quality and nutrition maintenance, solar drying is best for low-cost and more valuable products.

 Ascorbic acid, also known as Vitamin C, defines the nutritional quality of fruits, and its amount is affected by postharvest handling (Hamed et al., 2019). Thermal oxidation during processing and successive oxidation in storage are the reasons for ascorbic acid reduction (Lee and Kader, 2000). Rashmi et al., (2005) revealed lower levels of vitamin C in the storage of oven dried slices (Gupta, 2007). Ascorbic acid in tomato drying is also reduced as it is one of the heat-affected constituents of food products (Hussein et al., 2016). Ascorbic acid, being sensitive to heat, declines with drying time and temperature enhancement. Hussein et al., (2016) also recorded vitamin C changes due to drying methods that affect the physiochemical features (Correia et al., 2015). It showed that drying methods have significant effects on vitamin C content. Vit C breaks quickly when exposed to heat as thicker slices get more time to dry, but lower vit C was observed rather than thinner slices, where more vitamin C breakage was seen (Marwati, 2017).

Thicker slices get more time to dry, but more ascorbic acid was observed due to the lower temperature. Ramallo and Mascheroni (2004) also observed such a trend during pine apple drying in okra (Pendre et al., 2012). Decline occurs due to temperature, drying time, and enzyme activity, and lower vit C was observed in sun-dried samples as compared to oven dried samples (Simanjuntak, 2009), which is in line with Panceri et al., (2013) results. A similar trend was noted by others (Evrendilek, 2016). The sensitivity of ascorbic acid to heat, oxygen, and light, varying drying times, and drying conditions in natural sun-dried fruits require more drying time (Hussain et al., 2013). The highest quantity was reported in mechanical cabinet dryers, followed by solar cabinet dryers and sun drying (Bhat et al., 2014).

Non-reducing sugars decline as the time of storage is extended due to the presence of strong oxidizing agents that convert them to simple sugars, although they cannot hydrolyze easily (Sofy et al., 2022). Acidity increases with the decline of non-reducing sugars (Ruiz et al., 1997). Kim et al., (2014) dried samples in the oven, open sun, and solar dryer, which showed that extender drying time in the oven and open sun caused amino acid and reducing sugar decline that led to the browning reaction known as the Millard browning reaction, which causes brown pigmentation of products, rather than solar dried samples.

Reducing sugars are composed of simple sugars (glucose and fructose + aldehyde group) that have the ability to decrease other substances (Kamal and Rabeh, 1989, Fatima et al., 2023). Reducing sugar increased due to the conversion of starch into carbon dioxide and polysaccharides. Furthermore, the metabolic breakdown of carbon dioxide and polysaccharides leads to the production of water-soluble sugar (Singh et al., 2017a, Singh et al., 2017b). Increases in reducing sugars occur due to polysaccharide hydrolysis and non-reducing sugars conversion to reducing sugars. Similar results were observed in mango slices (Amitabh et al., 2000), pumpkin candy (Muzzaffer, 2006), dehydrated fig (Naikwadi et al., 2010), and bamboo shoot candy (Synrem, 2013). Reducing sugar during storage is enhanced due to starch conversion into simple sugar (Ruiz et al., 1997, El-Mahdy et al., 2021), but it can be declined due to acidity, which has a great influence on reducing sugar in dried fruits. Sugars are enhanced in dried fruits due to water loss as compared to fresh commodities rather than drying methods effects (Sneha, 2018). Gopalan et al., (2007) reported significant enhancement in dried fruit chemical constituents except TA and pH, which were reduced. Aggarwal et al., (2010) recorded the highest reducing sugars in the arils of pomegranate dried by a solar collector compared to open sun drying. Non-reducing sugars decline due to dehydration, while reducing sugars get enhanced due to furfural formation and the inversion of non-reducing to reducing sugars due to heat and an acidic environment (Galal et al., 1997).

Water losses in commodities are reduced by dehydration, which increases shelf life with quality maintenance (Olveira-Bouzas et al., 2023). Solar dehydration is a cheap and effective method for commodity dehydration with round-the-year availability (Bourdoux et al., 2016). Microbial assessment is an important aspect of determining the microbial load in dried food, which could be acceptable and would improve the shelf life (Sarker et al., 2014). During the drying process, some microorganisms are destroyed but are not lethal to all microbes. More importantly, there might have been contamination during the processing period. The higher TSS and acidity of the product might have also played role in product maintenance (Chavan et al., 2010, Fatima et al., 2022).

Microbe population gets inhibited due to faster drying with sufficient nutritional quality maintenance (Mercer, 2014) rather than Sun drying due to the lengthy drying time for microbe spread (Lewicki et al., 2002), although it is a cheap method (Leon et al., 2002). Furthermore, pathogen attacks, ambient dust cause quality loss (Aghbashlo et al., 2009, Ashry et al., 2018) as compared to solar and osmo dehydration due to a controlled environment (Doymaz and Ismail, 2011) and protection from atmospheric effects (Baradey et al., 2016). Due to the hygroscopic nature of dried fruits, protect them from microorganisms (Zhang, 2017), as they live in scarce water conditions too (Finn et al., 2013), which needs to be corrected by using advanced drying tools (such as solar collectors) (Hawaree et al., 2009). In such conditions, microbes get disturbed, which leads them to prevent cellular damage rather than repair it (Singh et al., 2017a, Singh et al., 2017b). In dried products, the osmolarity between intracellular and intercellular should be maintained. Solar drying has no disease incidence, which is cheap and also effective, while ovens have a microbial load within safe limits, but open sun is exposed to more microbes, leading to a high disease incidence (Archer et al., 1998).

Pectin degradation and moisture taken by slices resulted in taste changes during storage duration (Sharma, 2002, Mukhtar et al., 2022). The taste of dried peach samples decreased during the 90-day storage period, but at a very slow rate. During storage, taste changes occur due to sugar conversion and TA enhancement (Sing et al., 2012). During storage, taste is affected by carotenoid degradation and browning reactions (Dutta et al., 2006).

 Based on color consumers select and accept commodities, which makes it an important quality factor (Doymaz, 2017). Various chemical changes due to certain treatments could be observed in the color which is why it is a quality indicator factor, but the type of food packaging also affects its color (Echegaray et al., 2023), and it also changes during storage as a result of oxygen, non-enzymatic reactions, and SO_2_ reduction in slices (Sharma, 2002). Osmo dried samples maintain a color similar to fresh (Rodrigues et al., 2003), which was the result of sugars thin layered form on the slice surface that inhibits browning reactions that led to color changes (Rahman and Mujumdar, 2007). While inhibition of beneficial enzymatic changes in the oven led to dark color slices (Jha et al., 2021). Compared to these methods, solar drying is the best method as it maintains quality and is safe (Ratti, 2001). Pigment oxidation and degradation occur in open sun-dried samples, which are due to extended oxygen exposure along with maximum water loss (Topuz and Ozdemir, 2003). Similar observations were also recorded by Maurya et al., (2018).

Various studies have investigated TPC in horticultural commodities (Ji et al., 2012), especially in vegetables (Bukya et al., 2018). Phenols and antioxidants have a strong connection (Nunes et al., 2016). The amount of phenols in horticultural products depends on various factors (cultivar, growth circumstances, storage, transport, and handling expertise) (Bennett et al., 2011). Higher temperatures led to more phenol loss which partly depends on variety and climate, and vice versa (Yin et al., 2023). Different TPC loss ranges (higher and lower) have been found (Deng et al., 2019, Sakooei-Vayghan et al., 2020). 80 % of antioxidant activities are due to the combined action of vitamin C and phenols (Podsędek, 2007). Heating of commodities causes phenol degradation (oxidation, cleavage of covalent bonds, or speedy oxidation reactions), where its constancy is temperature- and time-dependent (Riehle et al., 2013). Drying causes TPC loss in any drying procedure (Suvarnakuta et al., 2011) due to polyphenol oxidase (PPO) activity (reduction of intermolecular reactions) (Bennett et al., 2011). Heating or non-heating processes led to phytochemicals (either bound or free) degradation (structure alteration), with new efficient antioxidant formation. Phenol structure alteration (Yellow and brownish pigment formation or phenols binding to other compounds) or reduction occurs as a result of certain food processes (Clifford, 2000), such as the Maillard reaction (Martin-Cabrejas et al., 2009).

Speedy slice dehydration in the solar collector showed a lower reduction in phenols (high bound phenolic release from breakdown of cellular components) as a result of lower PPO activity (Vega-Galves et al., 2009). Solar collectors have qualitatively dried slices. Oxygen captivation through the solar collector led to PPO oxidation (Korus, 2011). Open sun drying (more dehydrating time) causes more PPO activity, which leads to a reduction in heat-sensitive TPC along with enzyme inactivation (Sidhu et al., 2022). Lower exposure with less TPC reduction in oven drying happened than open sun drying. Browning and phenol loss occur as a result of oven or open-sun drying (Babu et al., 2018). A reduction in TPC occurs with slice thickness; higher TPC is reported in thicker slices than thinner slices (Ibrahim et al., 2021). TPC plays a great part in human health. Phenols decline with the passage of time in storage might be due to autoxidation (Deng et al., 2019), where they are not dependent on light and temperature during storage (Udomkun et al., 2016). But it strongly depends on temperature and storage period, as oxidation and polymerization lead to phenol reduction (Kapoor and Aggarwal, 2015). A similar downward trend was observed by other studies too (Korus, 2011, Li-Zhen et al., 2022).

Flavonoids which have the largest natural phytophenols (El-Beltagi et al., 2018), depend on cultivar, cultural tools, and climate (Alasalvar and Shahidi, 2013, El-Beltagi et al., 2019). Being secondary metabolites of polyphenols, flavonoids have roles as antioxidants (Arifin and Ibrahim, 2018), and chelating agents (Heim et al., 2002). It also helps in cancer and heart disease reduction (Crozier et al., 1997, El-Beltagi et al., 2023b). Furthermore, flavonoids could help improve human health through horticultural product consumption (Ignat et al., 2011).

Flavonoids (reducing agents, hydrogen donors, or singlet oxygen quenchers) have chelating ability along with strong antioxidant activity as a result of high redox capacity (Tsao and Yang, 2003). More exposure time and temperature led to flavonoids decline (Schieber et al., 2001), although they were stable at high temperatures (Papoutsis et al., 2017). Open-sun dehydration led to a 91–96 % lower decline of flavonoids than oven and open-sun drying (Ferreira et al., 2002), depending on the particular flavonoid’s constancy and sugar existence in their molecule (Buchner et al., 2006, Ali et al., 2011). Reduction of flavonoids occurs in every drying procedure (Kamiloglu et al., 2014). The decline of flavonoids occurs in storage (Carbone et al., 2009), which should be retained using effective food processing and storage components to avoid loss and detrimental chemical changes (Vidinamo et al., 2022). More flavonoids and phenols decline with high storage temperatures and storage periods (Song et al., 2022).

Antioxidants (carotenoids, phenols, alkaloids, nitrogenous compounds, or organosulfur substances) (Afify et al., 2012, El-Beltagi et al., 2022b) have a strong connection with phytochemicals (Zielinska and Zielinska, 2019, Nguyen et al., 2020). Phenols and AA quantity are enhanced or retained based on product type, not on dehydration method. A fall in AA activities occurs due to various factors (degradative enzymes, phytochemical loss upon drying, and loss of antioxidant enzyme activities) (Chan et al., 2012, Frank et al., 2022). Altered influences of drying have been noted in AA activities in vegetables and fruits (Kuljarachanan et al., 2009).

Phenol loss upon drying leads to antioxidant enhancement or fall (Li et al., 2007; Ahmad et al., 2021). The quantity of AA declines upon drying but could be lower or retained through the formation of new efficient antioxidant substances (Anese et al., 1999). Similarly, the fall of AA and phenols is also due to other bioactive features (Deng et al., 2019). The best drying tool results in less phenol reduction with high AA activity in various commodities (Anwar et al., 2012). Our results are strongly in accordance with Chan et al., (2009), who noted more antioxidants in the open sun while fewer in oven drying. Volatile compounds release and reduce antioxidants as a result of lengthy exposure time to heat compared to a shorter time in oven drying (Nguyen et al., 2020), along with extra phenol formation and speedy PPO inactivation (Chan and Lim, 2006).

Finally, oxidation reduction led to lower antioxidant activities (Chan et al., 2012). Higher antioxidant losses occur in open sun drying due to the higher temperature than oven drying with less drying time (Lukinac and Jukić, 2022). Longer drying time with a higher temperature (Perez-GiAlvez et al., 2005) led to the lowering of certain substances (caramelization, Maillard reactions, enzymatic reactions, pigment degradation, or L-ascorbic acid oxidation) (Rufián-Henares et al., 2013). Thinner slices represented lower antioxidant activities than thicker slices due to enhancement of other biochemical compounds (El Mouzahim et al., 2023). Solar drying is the safest method in the case of sensory and biochemical compound maintenance (Mongi et al., 2015). Lengthy storage periods led to AA decline (Deng et al., 2019), while chemical and enzymatic activities led to enhancement (Bennett et al., 2011).

## 5 Conclusion

In conclusion, the higher drying time, moisture content, and drying rate of peach slices were recorded in solar collector with a 0.5 cm slice thickness, followed by 1 cm and 1.5 cm, respectively. Drying methods, slice thickness, and storage duration significantly influenced all studied attributes. Moreover, most of the two-way interactions were significant, while the three-way interactions for all studied attributes were non-significant. Peaches sliced at 0.5 cm thickness and dried in a solar collector stored for 90 days retained their total soluble solids, titratable acidity, TSS-acid ratio, ascorbic acid, reducing and non-reducing sugars, and antioxidant activity. While maximum phenolic and flavonoid content was recorded in slices of 1.5 cm thickness dried by the solar method, peach slices with a thickness of 1.5 cm did not minimize the disease incidence because of their poor taste and color. Better taste and color with minimum disease incidence were recorded in peach slices dried in a solar collector with 0.5 cm thickness up to 90 days of storage.

Authorship contribution statement.

Hossam S. El-Beltagi, Ayesha Khan, Syed Tanveer Shah: contributed equally to this work.

Hossam S. El-Beltagi, Ayesha Khan, Syed Tanveer Shah, Abdul Basit, Muhammad Sajid, Muhammad Hanif and Heba I. Mohamed: performed the overall experiment and data analysis. Hossam S. El-Beltagi, Ayesha Khan, Syed Tanveer Shah, Abdul Basit, Muhammad Sajid, Muhammad Hanif and Heba I. Mohamed: performed experiments to confirm results and wrote the manuscript together. Hossam S. El-Beltagi, Ayesha Khan, Syed Tanveer Shah, Abdul Basit, Muhammad Sajid, Muhammad Hanif and Heba I. Mohamed: designed and managed whole experiments and finalized the manuscript.

## Funding

This work was also supported by Deanship of Scientific Research, Vice Presidency for Graduate Studies and Scientific Research, King Faisal University, Saudi Arabia (GRANT 4283).

## Declaration of competing interest

The authors declare that they have no known competing financial interests or personal relationships that could have appeared to influence the work reported in this paper.
